# Supplementary material for: Mapping and annotating genomic loci to prioritize genes and implicate distinct polygenic adaptations for skin color
Source: Nat Commun. 2024 Jun 7;15:4874. doi: 10.1038/s41467-024-49031-4 (PMC11161515; doi:10.1038/s41467-024-49031-4)
Supplement: Supplementary file 1 — Supplementary Information [file 41467_2024_49031_MOESM1_ESM.pdf]

Supplementary Information

Supplementary Note

Genotype Quality Control (QC)

Supplementary Figures

Supplementary Fig. S1. Correlation between skin color and covariates in 40,790 unrelated participants.

Supplementary Fig. S2. Distribution of sun exposure variables across different age groups.

Supplementary Fig. S3. Principal component analysis of genetic variants.

Supplementary Fig. S4. Principal component analysis of genetic variants in East Asian population.

Supplementary Fig. S5. Quantile-quantile plots of GWAS results.

Supplementary Fig. S6. Correlation between the effect size estimates in the discovery GWAS and variant loadings of the first 10 PCs.

Supplementary Fig. S7. Manhattan plots depicting the association between skin color traits and variants on chromosome X.

Supplementary Fig. S8. Regional plots of GWAS results for the (**a**) *GLIS1*, (**b**) *OCA2*, and (**c**) *MC1R* loci.

Supplementary Fig. S9. GWAS of categorical skin color using POLMM.

Supplementary Fig. S10. Comparison of the lead variants in the GWAS with discovery (*x*-axis) and replication set (*y*-axis).

Supplementary Fig. S11. Comparison of the lead variants from a 10-fold cross-validation of the GWAS for (**a**) L^*, (**b**) a^*, and (**c**) b^*.

Supplementary Fig. S12. The power adjusted transferability (PAT) ratio of the lead variants in replication cohort and 10-fold cross-validation of GWAS.

Supplementary Fig. S13. The number of significant loci that increases with increasing sample size in the permutation meta-analysis.

Supplementary Fig. S14. The median proportion of significant loci that increases with increasing sample size in the permutation meta-analysis.

Supplementary Fig. S15. The relative effect size for skin color traits for each quintile group of polygenic score.

Supplementary Fig. S16. Functional enrichment analysis of skin color trait-associated loci.

Supplementary Fig. S17. Regional plots of (**a**) GWAS results near *SPIRE2* and *MC1R* and (**b**) eQTL results of colocalized (PP.H4.abf > 0.7) genes near the corresponding loci.

Supplementary Fig. S18. Colocalization results.

Supplementary Fig. S19. Number of colocalized genes (PP.H4 > 0.8) in each tissue.

Supplementary Fig. S20. Batch effect correction using Harmony and gene expression patterns of skin cell-type markers.

Supplementary Fig. S21. Signals of polygenic adaptation for *b** and *a** across 1000 Genomes Project populations.

Supplementary Fig. S22. Interplay between polygenic score and sun exposure for (**a**) *L**, (**b**) *a**, and (**c**) *b**.

Supplementary Fig. S23. Normality of residuals for each skin color trait in a null model (a linear model with only covariates) assessed in each group (**a**) A and (**b**) B.

Software URLs utilized in this study

Supplementary Note

Genotype quality control (QC)

We performed QC in three steps prior to imputation: 1) first variant-level QC, 2) sample-level QC, and 3) second variant-level QC.

For the first variant-level QC, variants with a call rate < 98% and duplicate variants with the same base-pair position were excluded. Variants with MAF < 1% or showing deviation from Hardy-Weinberg equilibrium ($P < 1.0 \times 10^{-6}$) in unrelated samples from each population were excluded. The MAF calculation and Hardy-Weinberg equilibrium test of variants on chromosome X were performed only for female participants. Familial relationships among the study participants were estimated using KING (v.2.1)[1], and 8,018 individuals with related individuals with second-degree or closer relationships were excluded to construct unrelated samples for variant-level QC. Sample-level QC was performed using the variants that passed the first variant-level QC. Samples were excluded based on the following criteria: call rate < 95% (251 individuals were excluded), heterozygosity rate three standard deviations away from the mean (319 individuals were excluded), and discordance between the reported and inferred sex based on the heterozygosity rate on chromosome X (276 individuals were excluded). After excluding these low-quality samples, second variant-level QC was performed using procedures identical to those used for the first QC with raw genotype data. In addition, variants that showed significant associations ($P < 5.0 \times 10^{-8}$) with groups A and B were excluded to minimize the false positives due to batch effect. Associations of variants with each pair of genotype batches were tested using a logistic mixed model implemented in SAIGE (v.0.35.8)[2].

Our samples were projected onto the principal component analysis plot of the 1000 Genomes Project phase 3 using eigenvectors from the 1000 Genomes Project samples (**Figure S4**). The Korean and Chinese participants in the present study overlapped with the cluster of East Asians in the 1000 Genomes Project, although the study participants from undefined populations were relatively distant from the East Asian cluster. In addition, related samples were present among the study participants; therefore, we used a linear mixed model in the association analysis to adjust for population stratification and relatedness.

References

1. Manichaikul, A. *et al.* Robust relationship inference in genome-wide association studies. *Bioinformatics* **26**, 2867-73 (2010).

2. Zhou, W. *et al.* Efficiently controlling for case-control imbalance and sample relatedness in large-scale genetic association studies. *Nat Genet* **50**, 1335-1341 (2018).

Supplementary Figures

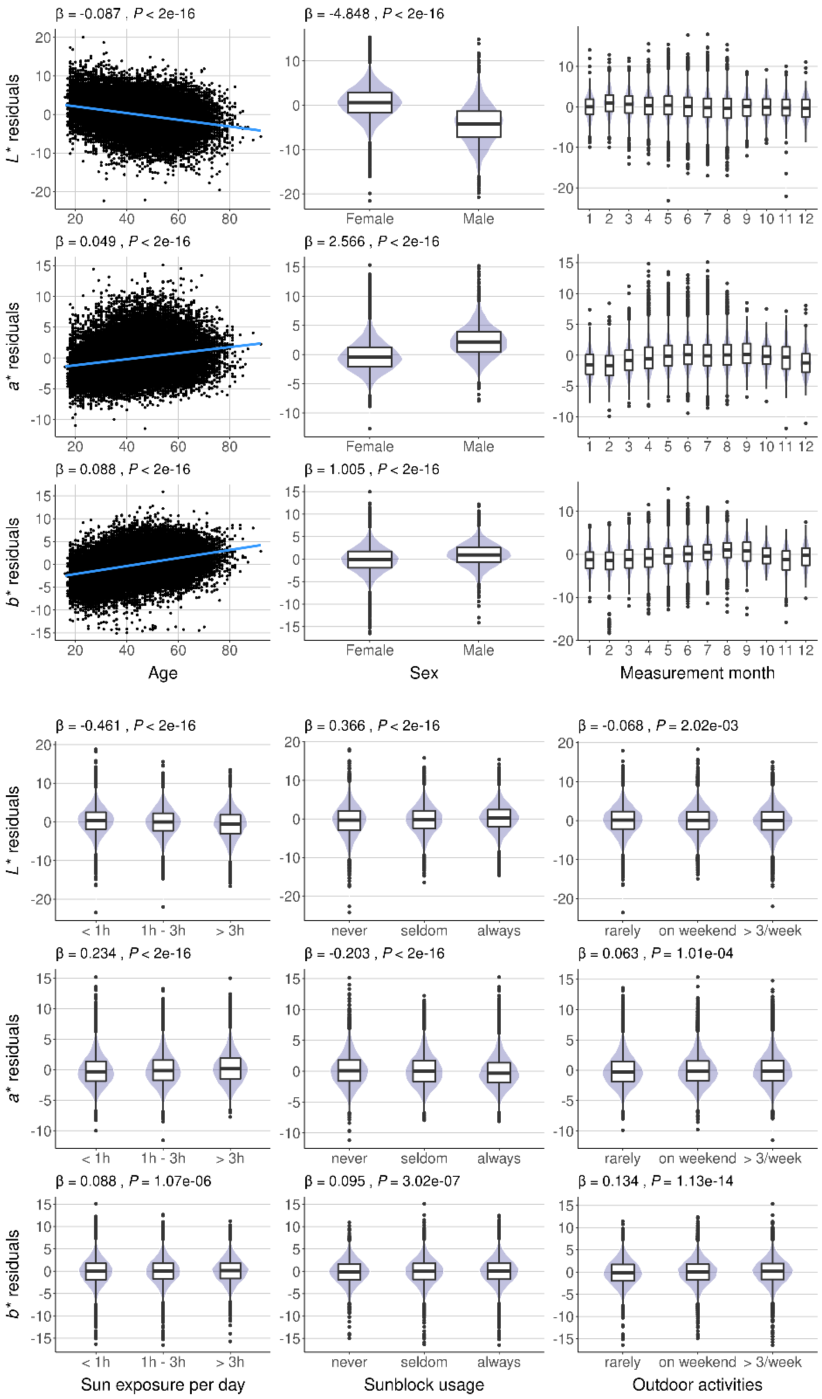

Supplementary Fig. S1. Correlation between skin color and covariates in 40,790 unrelated participants. Prior to correlation analysis, the residual values (*y* axis) of each skin color were obtained using a linear regression model adjusted for other covariates (*x* axis). Correlations between skin color and a continuous covariate, age, are demonstrated using a regressed line (blue lines) in a scatter plot. Other categorical covariates are presented as boxplots. Each plot presents the beta coefficient (β) and P-value (*P*) from a linear regression model of the residual skin color (*y* axis) with the described covariate (*x* axis) as explanatory variables.

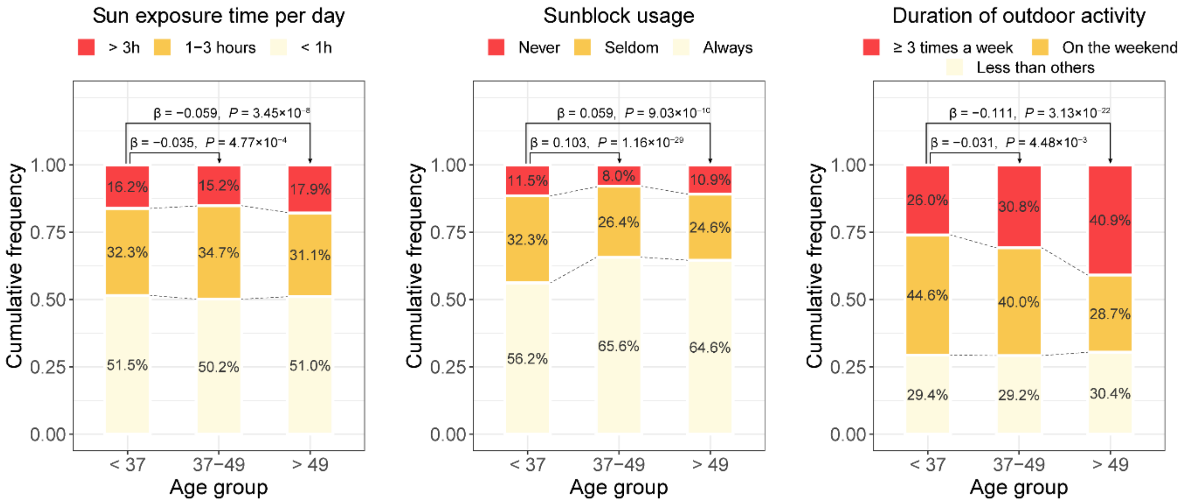

Supplementary Fig. S2. Distribution of sun exposure variables across different age groups. The proportion of individuals for each sun exposure variable within each age group (young age [< 37 years], middle age [37 - 49 years)] and old age [> 49 years] groups) is presented in the bar plot. Relative effect size (β) and *P*-value (*P*) of each age group for sun exposure variable are presented above the bar plot. The relative effect size was estimated using a linear regression model adjusted for the same covariates as in the GWAS.

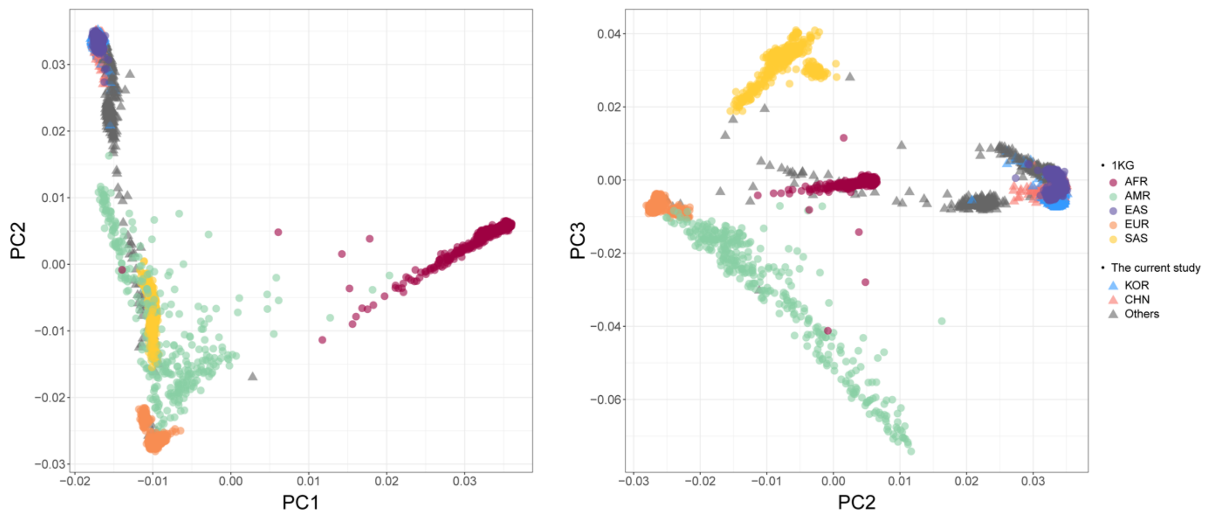

Supplementary Fig. S3. Principal component (PC) analysis of genetic variants. The first three PCs of genetic ancestry are presented. Each dot represents an individual from a colored ethnic group. Principal component analysis was conducted using the 1000 Genomes Project phase 3 samples (circles: AFR, AMR, EAS, EUR, and SAS) and the PCs of the current study samples (triangles: KOR, CHN, and others) were calculated using the eigenvectors from the 1000 Genomes Project phase 3. Abbreviations: AFR, African and African American; AMR, Latin American; EAS, East Asian; EUR, European; SAS, South Asian; KOR, Korean in the current study; CHN, Chinese in the current study.

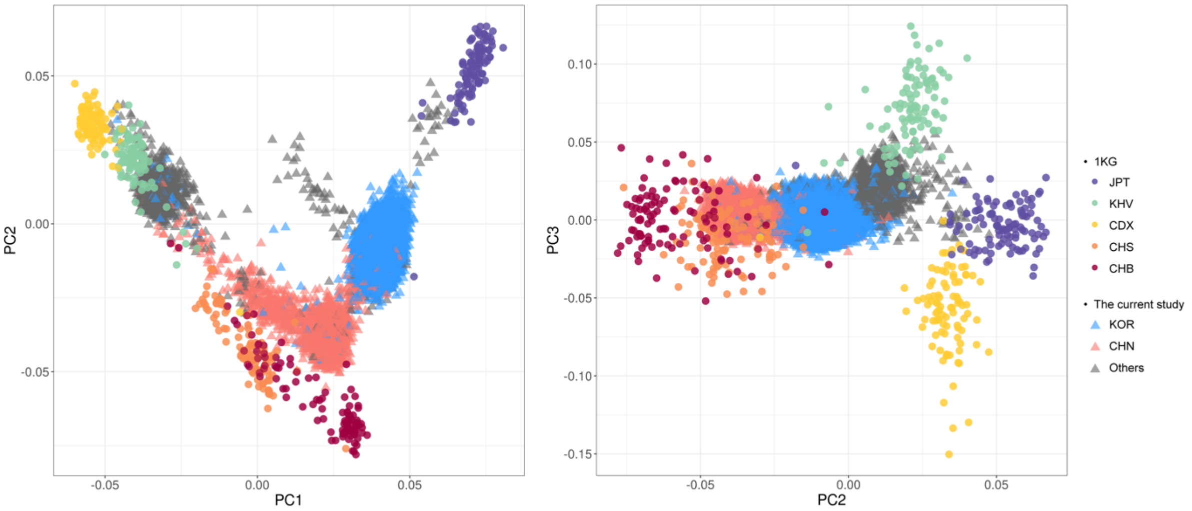

Supplementary Fig. S4. Principal component (PC) analysis of genetic variants in East Asian population. The first three PCs of genetic ancestry are presented. Each dot represents an individual from a colored ethnic group. Principal component analysis was conducted using East Asians from the 1000 Genomes Project phase 3 samples (circles: CDX, CHB, CHS, JPT, and KHV) and the PCs of the current study samples (triangles: KOR, CHN, and others) were calculated using the eigenvectors from the 1000 Genomes Project phase 3.

Abbreviations: CDX, Chinese Dai in Xishuangbanna, China; CHB, Han Chinese in Beijing, China; CHS, Han Chinese in Southern China; JPT, Japanese in Tokyo, Japan; KHV, Kinh in Ho Chi Minh City, Vietnam; KOR, Korean in the current study; CHN, Chinese in the current study.

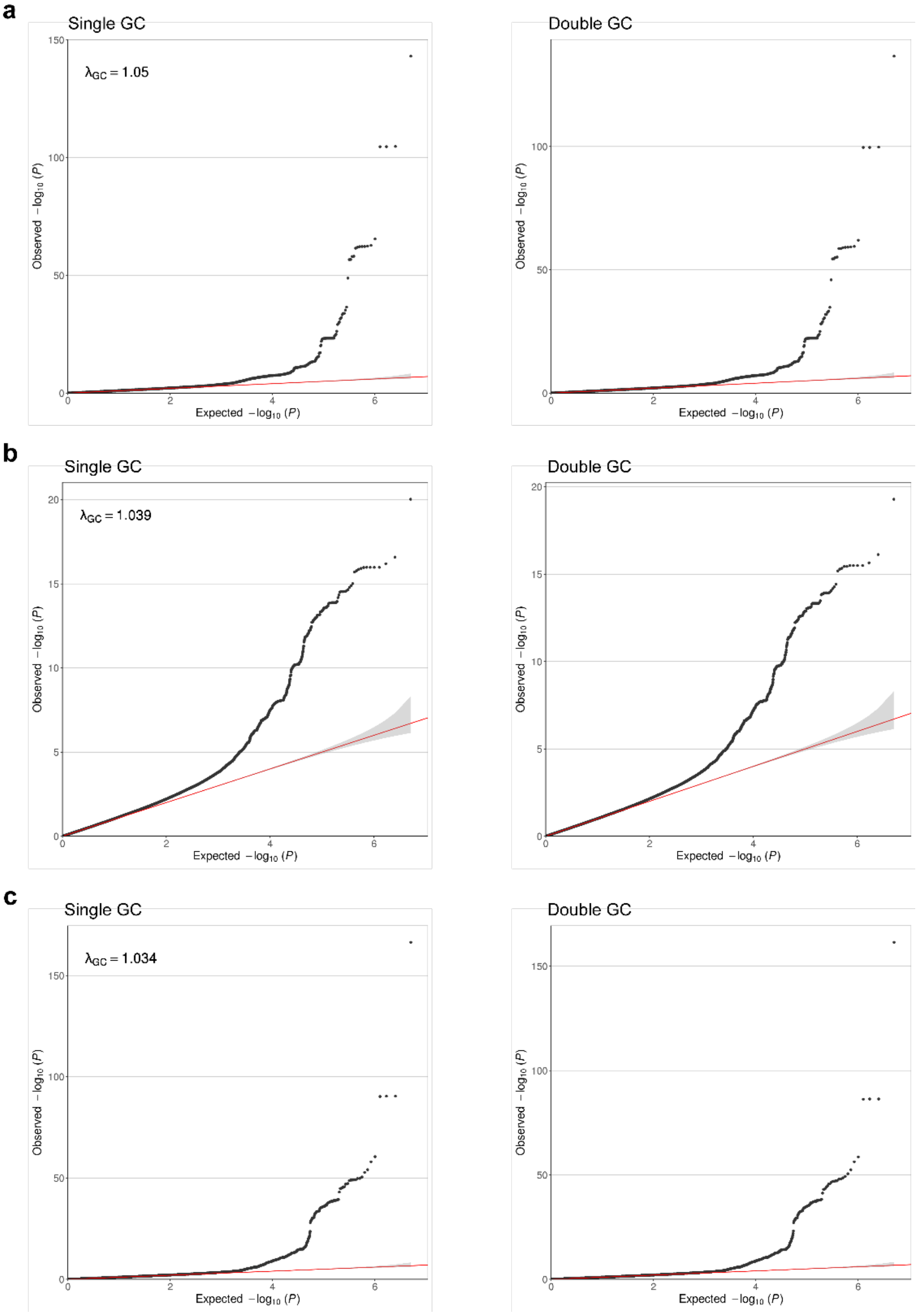

Supplementary Fig. S5. Quantile-quantile plots of GWAS results. Quantile-quantile plots of (**a**) *L**, (**b**) *a**, and (**c**) *b** GWAS results. Single genomic control results (left) and double genomic control results (right) are shown in the plots of each skin color trait. Negative logarithms of the observed (*y* axis) and expected (*x* axis) *P*-values are plotted for each SNP. The red line indicates the null hypothesis of no true association ($y = x$), and the gray region indicates the 95% confidence interval of the red line. The genomic inflation factor (λ_{GC}) before the second genomic control is shown in the upper left side of the single genomic control figure.

Abbreviations: GC, genomic control.

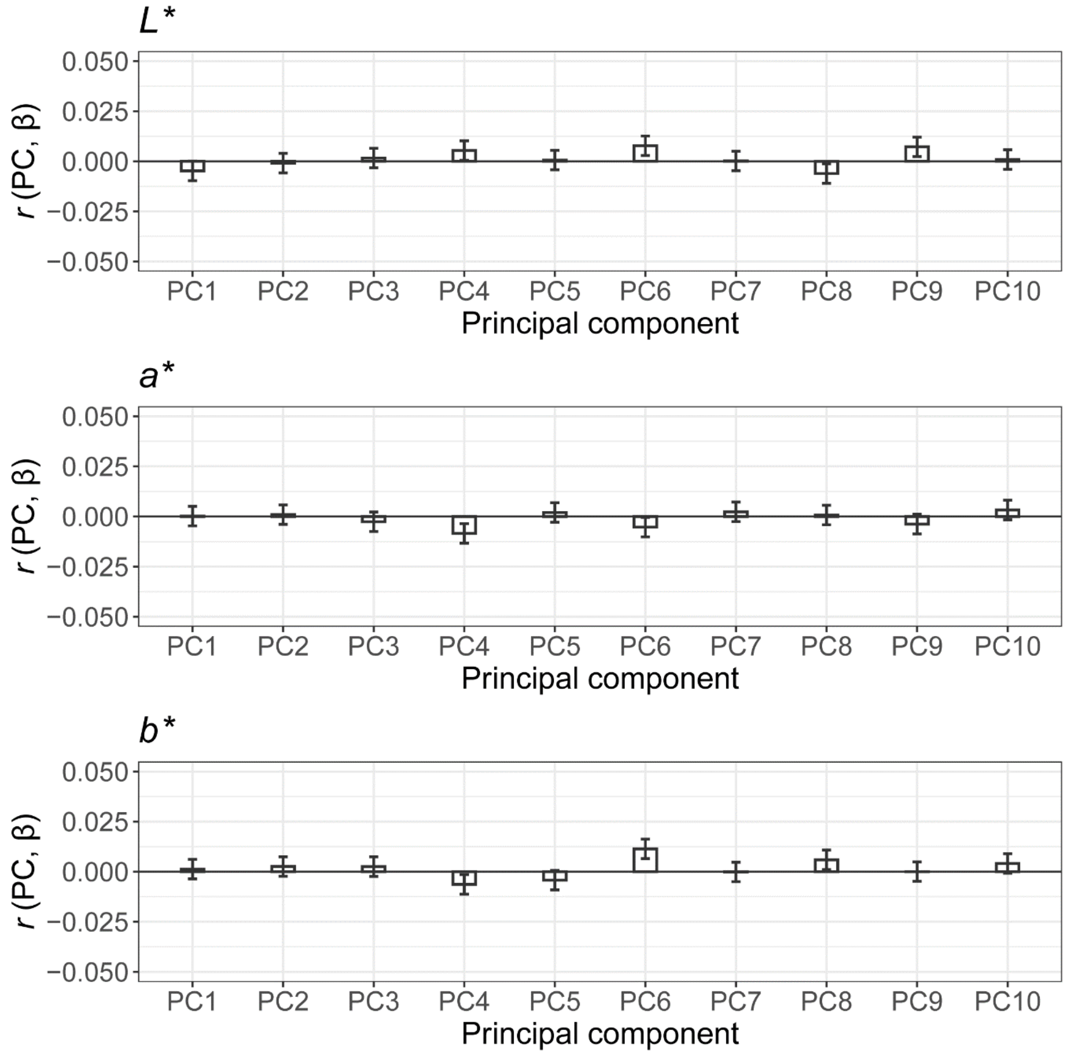

Supplementary Fig. S6. Correlation between the effect size estimates in the discovery GWAS and variant loadings of the first 10 PCs. Error bars indicate 95% confidence intervals for the Pearson's correlation coefficient.

Abbreviations: *r*(PC, β), Pearson's correlation coefficient between variant loadings and the effect size.

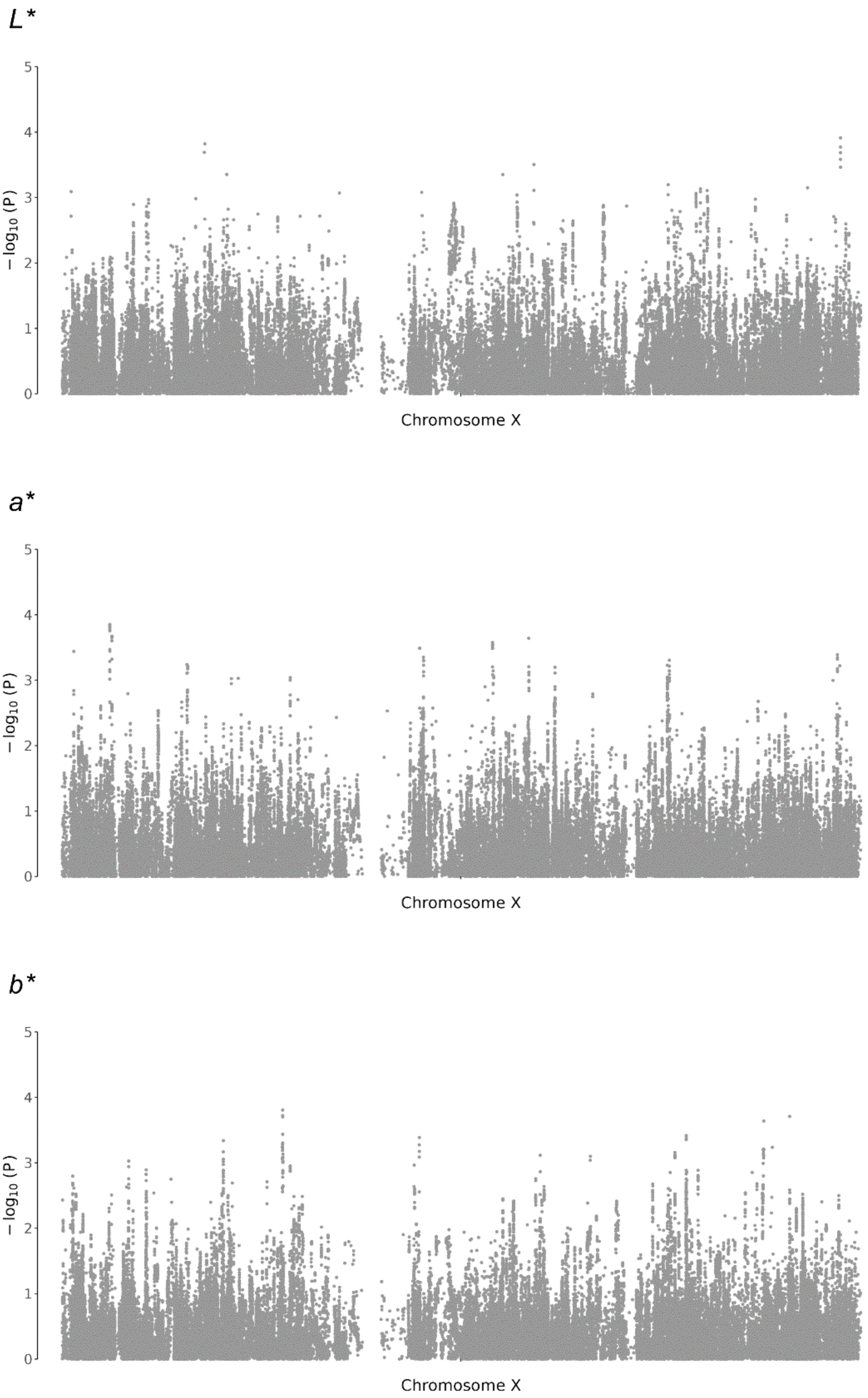

Supplementary Fig. S7. Manhattan plots depicting the association between skin color traits and variants on chromosome X. Each dot represents a variant plotted as -$\log_{10}$ (*P*) on the *y*-axis against the corresponding variant position on the *x*-axis.

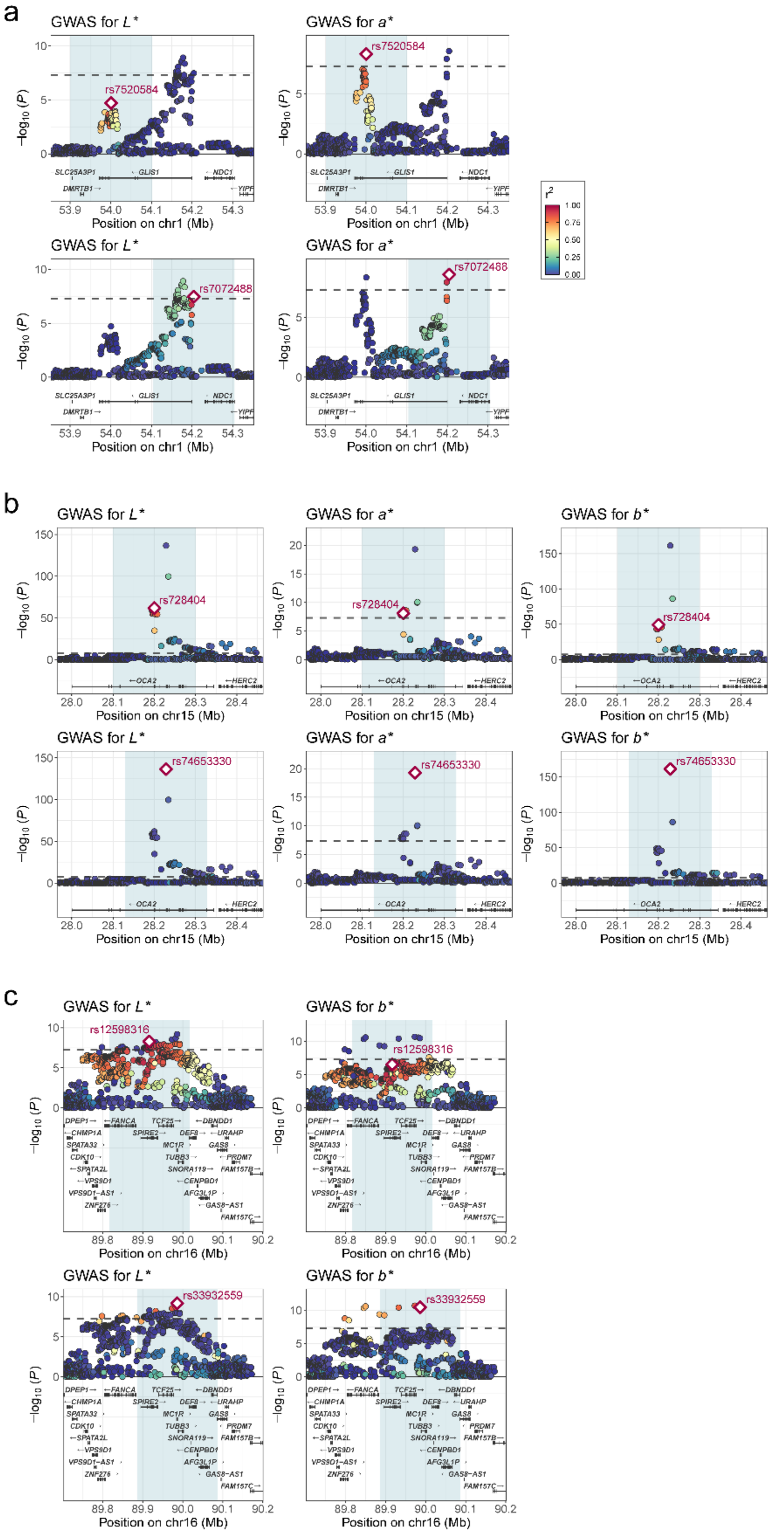

Supplementary Fig. S8. Regional plots of GWAS results on (**a**) *GLIS1*, (**b**) *OCA2,* and (**c**) *MC1R* loci, which are colored based on different lead variants; ±250 kb from lead variants in each locus. Each dot represents a variant plotted as -$\log_{10}(P)$ on the *y*-axis against the corresponding variant position (Mb) on the *x*-axis and is colored according to linkage disequilibrium with the lead variant (rhombus). The blue-shaded region was used for colocalization analysis (±100 kb).

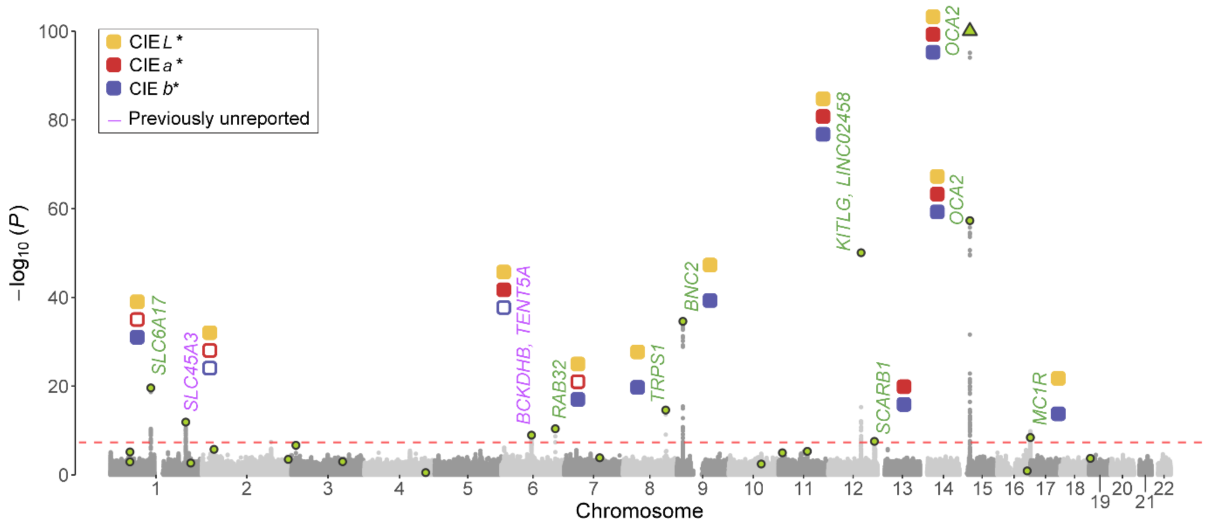

Supplementary Fig. S9. GWAS of categorical skin color using POLMM. The categorical skin color was classified based on individual typology angle (ITA°) value, as illustrated in **Fig. 1c**. Manhattan plot with -$\log_{10}(P)$ is presented for categorical skin color. The red horizontal line corresponds to the genome-wide significance threshold ($P = 5 \times 10^{-8}$). Green dots indicate the lead variants in the discovery GWAS of CIE LAB values. Genes in green and purple represent previously reported and unreported significant loci, respectively. Boxes in yellow, red, and blue represent significant loci of $L*$, $a*$, and $b*$, respectively, in the discovery GWAS of CIE LAB values; solid boxes indicate genome-wide significant loci, and boxes with colored borderlines indicate nominally significant loci ($P < 2.17 \times 10^{-3}$, Bonferroni's correction for 23 significant loci).

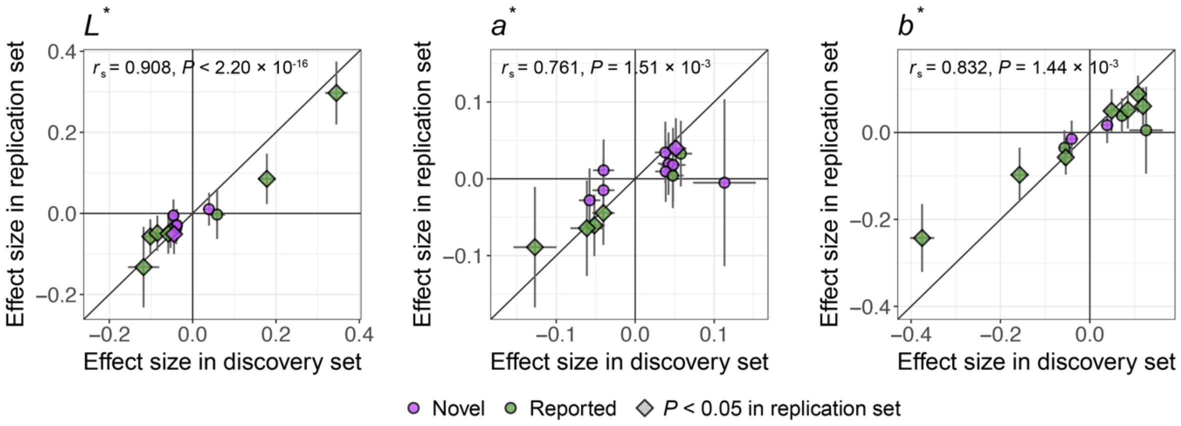

Supplementary Fig. S10. Comparison of the lead variants in the GWAS with discovery (*x*-axis) and replication set (*y*-axis). Green and purple dots indicate the lead variants at previously reported and unreported loci, respectively. Rhombus dots represent the lead variants with $P < 0.05$ in the replication set. Error bars indicate 95% confidence intervals for the effect size. Spearman's correlation (r_s) between effect sizes of lead variants is presented at the top of each plot.

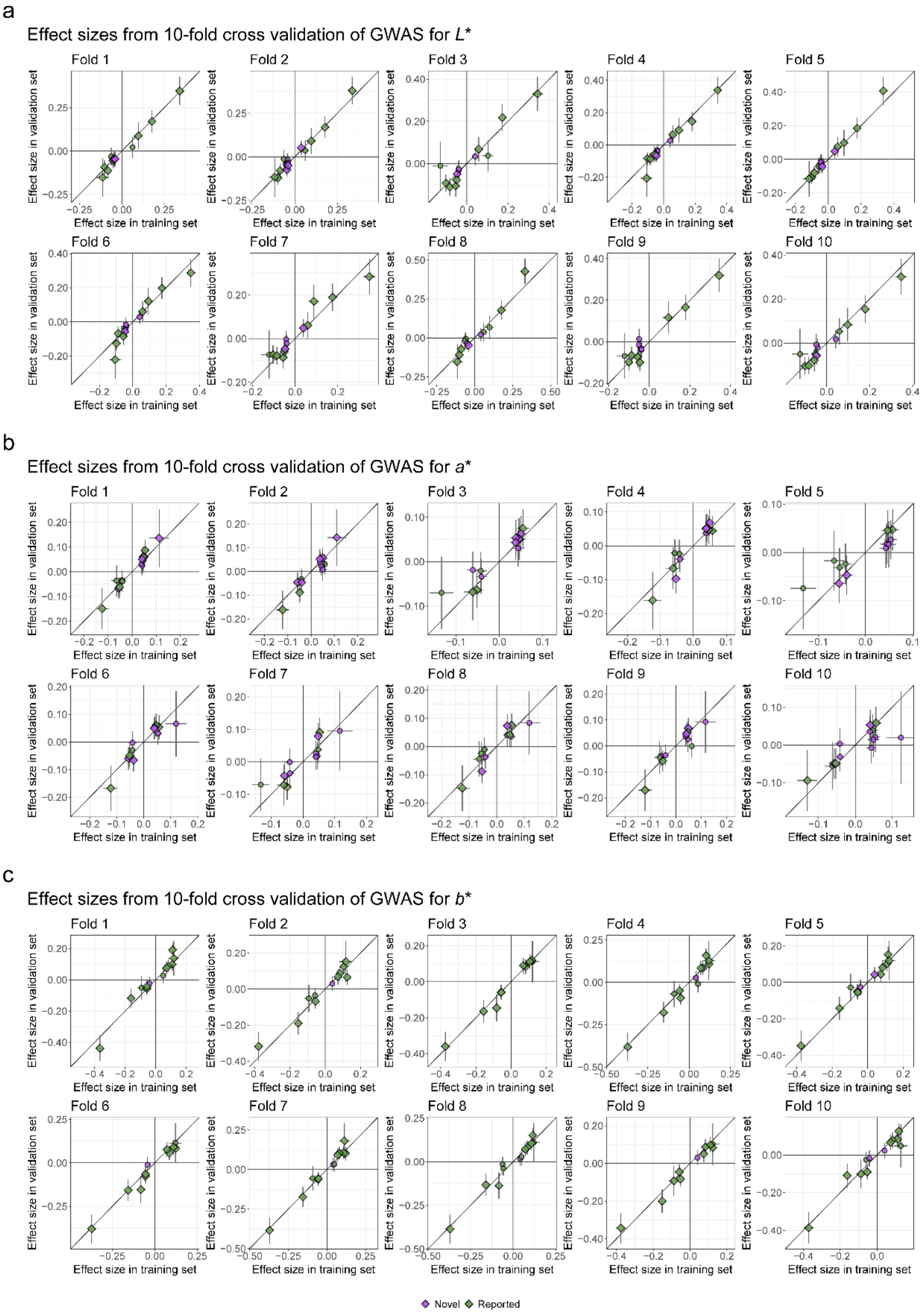

Supplementary Fig. S11. Comparison of the lead variants from a 10-fold cross-validation of the GWAS for (**a**) *L**, (**b**) *a**, and (**c**) *b**. The effect size from the GWAS with training (*x*-axis) and validation set (*y*-axis) in each fold are displayed. Green and purple dots indicate the lead variants at previously reported and unreported loci, respectively. Rhombus dots represent the lead variants with *P* < 0.05 in the replication set. Error bars indicate 95% confidence intervals for the effect size.

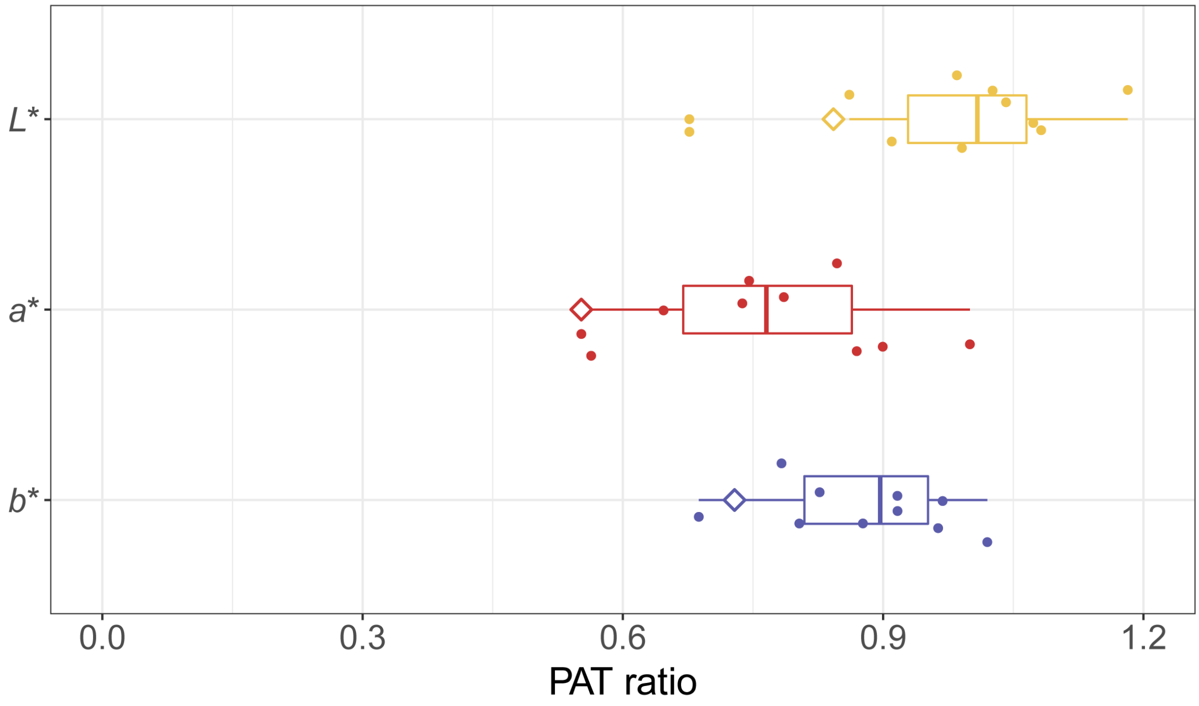

Supplementary Fig. S12. The power adjusted transferability (PAT) ratio of the lead variants in replication cohort and 10-fold cross-validation of GWAS. Each dot indicates a PAT ratio calculated on each fold of the cross-validation and a box plot shows the distribution of these ratios. A rhombus dot indicates a PAT ratio calculated from the replication cohort.

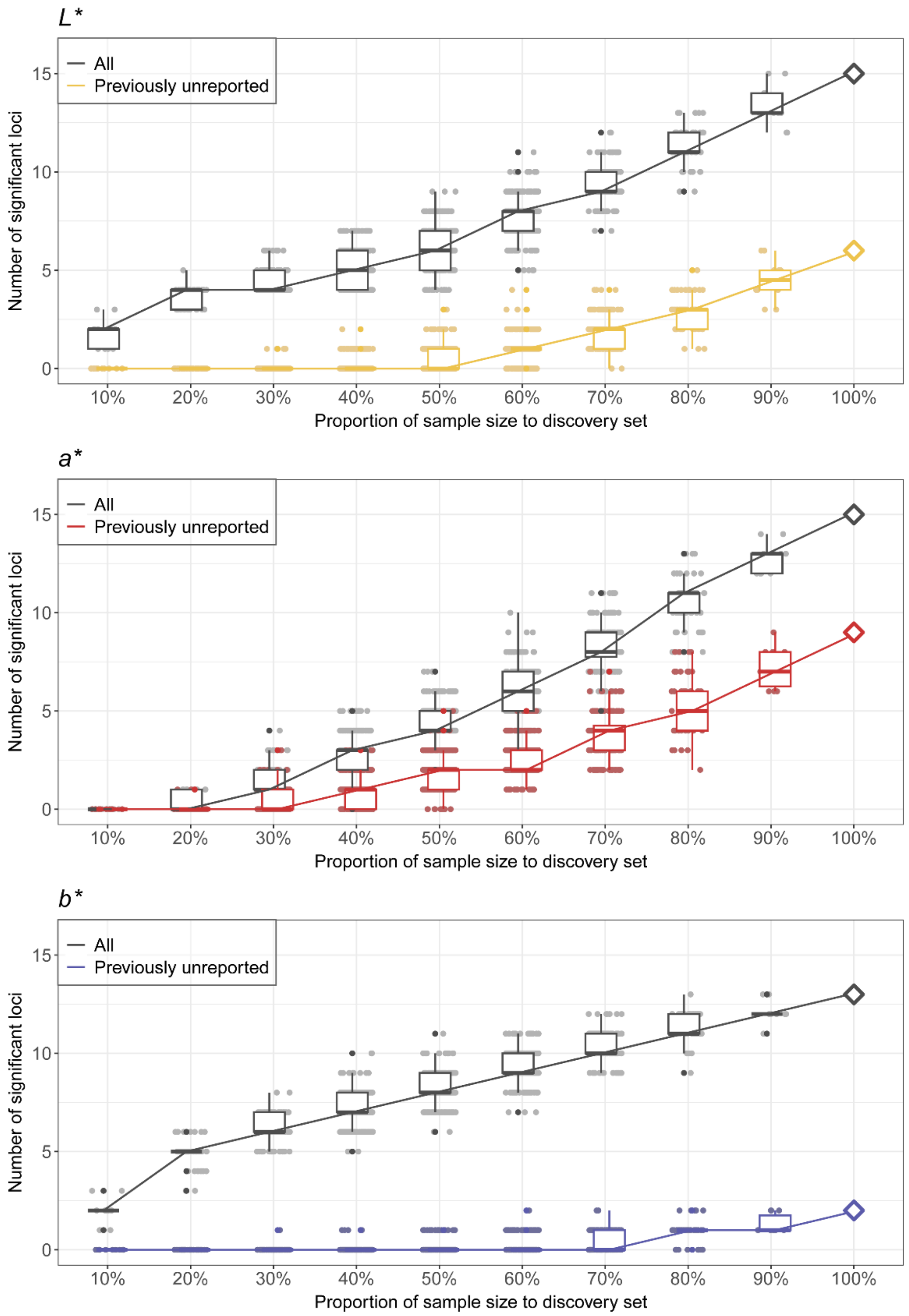

Supplementary Fig. S13. The number of significant loci that increases with increasing sample size in the permutation meta-analysis. Gray dots represent the number of significant ($P < 5.0 \times 10^{-8}$) loci (*y*-axis) obtained from GWAS with varying sample size (*x*-axis). Colored dots (*L**, yellow; *a**, red; *b**, blue) represent the number of significant unreported loci. Each box plot represents the distribution of number of significant loci with corresponding sample size and a line connects the median of each box plot.

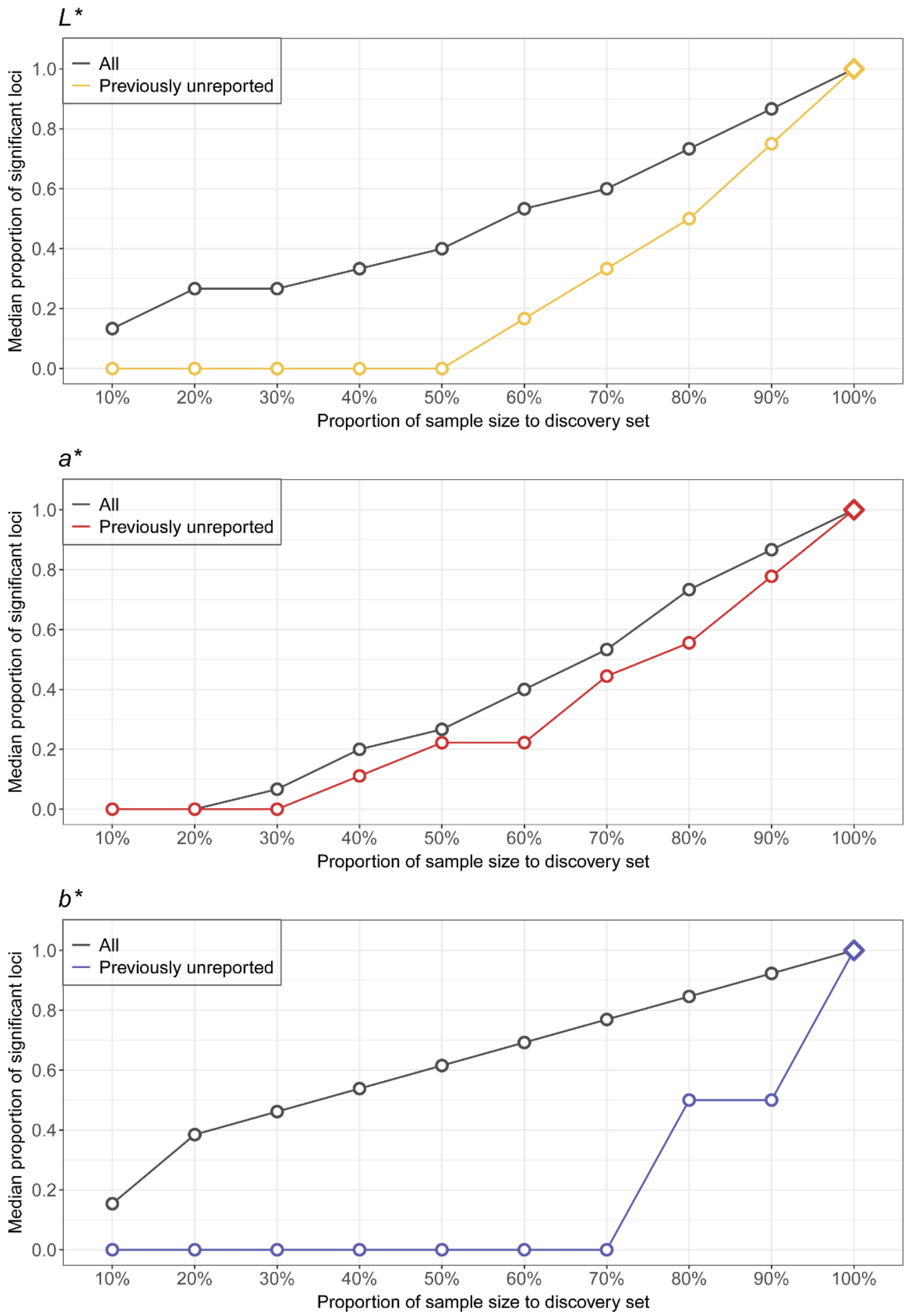

Supplementary Fig. S14. The median proportion of significant loci that increases with increasing sample size in the permutation meta-analysis. Gray (*L**, yellow; *a**, red; *b**, blue) dots represent the median proportion of significant ($P < 5.0 \times 10^{-8}$) loci (*y*-axis) obtained from GWAS with varying sample size (*x*-axis). Colored dots (*L**, yellow; *a**, red; *b**, blue) represent the median proportion of significant unreported loci.

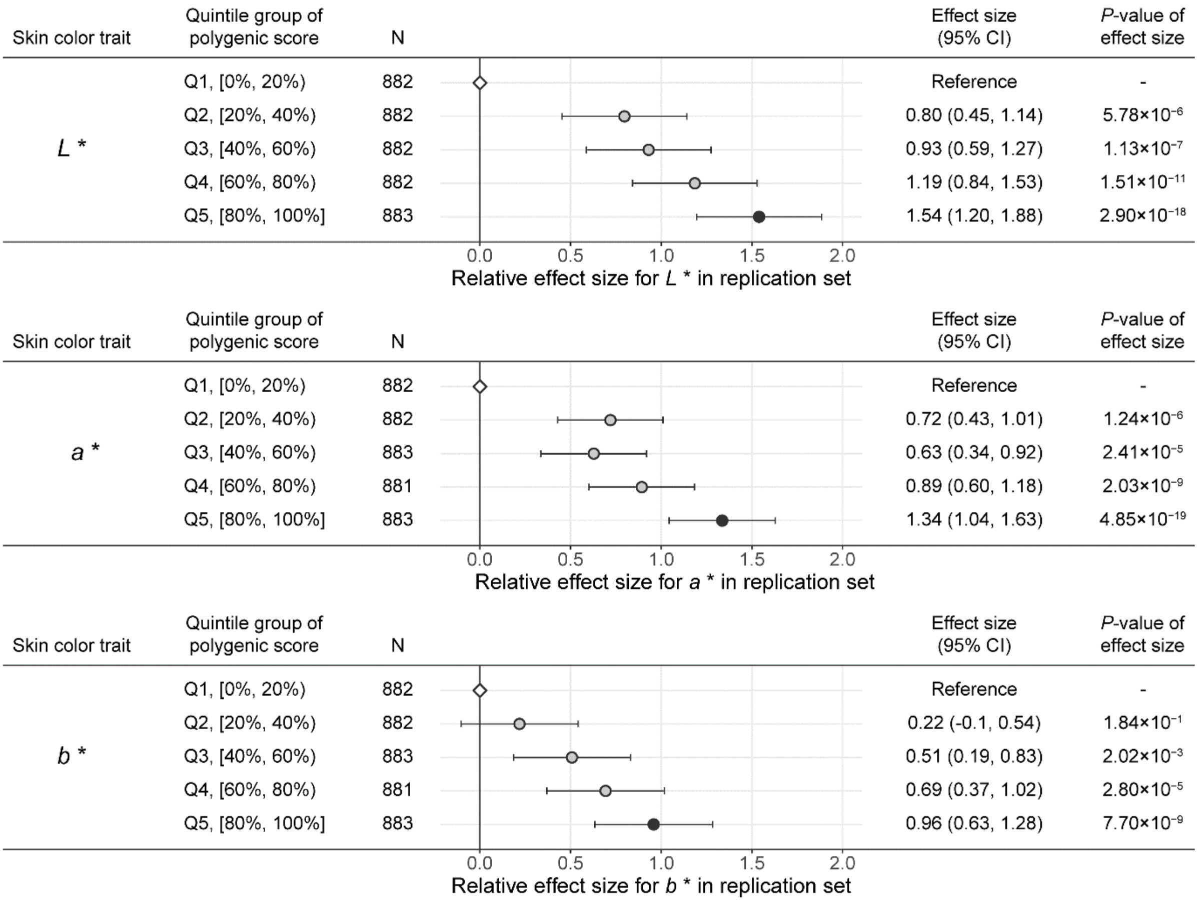

Supplementary Fig. S15. The relative effect size for skin color traits for each quintile group of polygenic score. A rhombic dot represents a reference group. Each dot represents the relative effect size. Error bars indicate 95% confidence intervals for the relative effect size.
Abbreviations: N, sample size; CI, confidence interval.

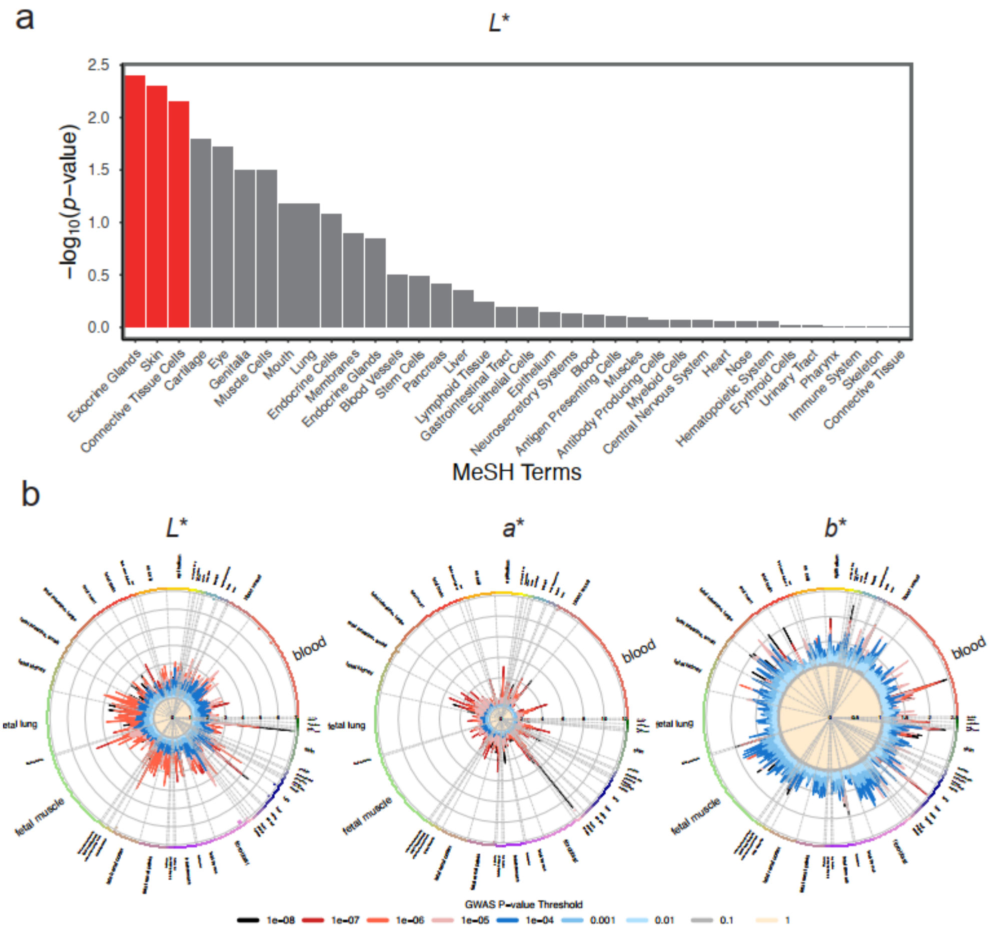

Traits	Tissue	Cell Type	GWAS P-Thresh	OR	*P*
*L**	Blood	Mobilized CD34 Primary Cells	1×10^{-5}	3.67	5.70×10^{-6}
*L**	Blood	Mobilized CD34 Primary Cells	1×10^{-5}	3.19	5.98×10^{-5}
*L**	Bone	Osteobl	1×10^{-5}	3.56	8.76×10^{-6}
*L**	Breast	HMF	1×10^{-5}	3.33	2.98×10^{-5}
*L**	Fetal renal cortex	Fetal Renal Cortex	1×10^{-6}	4.42	7.47×10^{-5}
*L**	Fibroblast	Penis Foreskin Fibroblast Primary Cells	1×10^{-5}	4.50	1.90×10^{-7}
*L**	Fibroblast	Penis Foreskin Fibroblast Primary Cells	1×10^{-6}	4.23	8.61×10^{-5}
*L**	Fibroblast	Penis Foreskin Fibroblast Primary Cells	1×10^{-5}	3.95	1.48×10^{-6}
*L**	Fibroblast	Penis Foreskin Fibroblast Primary Cells	1×10^{-4}	3.47	9.68×10^{-10}
*L**	Foreskin	HFF-Myc	1×10^{-5}	3.30	2.90×10^{-5}
*L**	Liver	Stellate	1×10^{-5}	3.79	2.16×10^{-6}
*L**	Lung	IMR90	1×10^{-6}	4.61	2.91×10^{-5}
*L**	Skin	Penis Foreskin Melanocyte Primary Cells	1×10^{-6}	4.75	3.00×10^{-5}
*L**	Skin	Penis Foreskin Melanocyte Primary Cells	1×10^{-5}	3.66	7.01×10^{-6}
*a**	gingival	HGF	1×10^{-8}	11.02	7.67×10^{-5}
*a**	blood vessel	AoSMC	1×10^{-5}	3.24	4.50×10^{-5}
*a**	fetal renal pelvis	Fetal Renal Pelvis Right	1×10^{-5}	3.14	5.37×10^{-5}
*a**	fetal intestine, small	Fetal Intestine Small	1×10^{-5}	3.09	5.97×10^{-5}

Supplementary Fig. S16. Functional enrichment analysis of skin color trait-associated loci. **a**, A bar plot is presented with the -$\log_{10}$(*P*-value) of the functional enrichment analysis results using DEPICT for the GWAS of *L**. Red bars indicate significant tissue types that reached a false discovery rate (*FDR*) of < 0.1. *FDR* significance was calculated using 36 MeSH terms. **b**, Functional enrichment analysis results obtained using GARFIELD for the GWAS of *L**, *a**, and *b** are presented as circular plots and tables. Radial lines represent the odds ratios of each cell type at nine GWAS *P*-value thresholds ($T < 1$ to $T < 10^{-8}$). The outermost circle represents the cell types colored by tissue type, and the tissue names are shown outside the circle. The dots inside the outermost circle indicate significance. Significant cell types are listed at the bottom of the table.

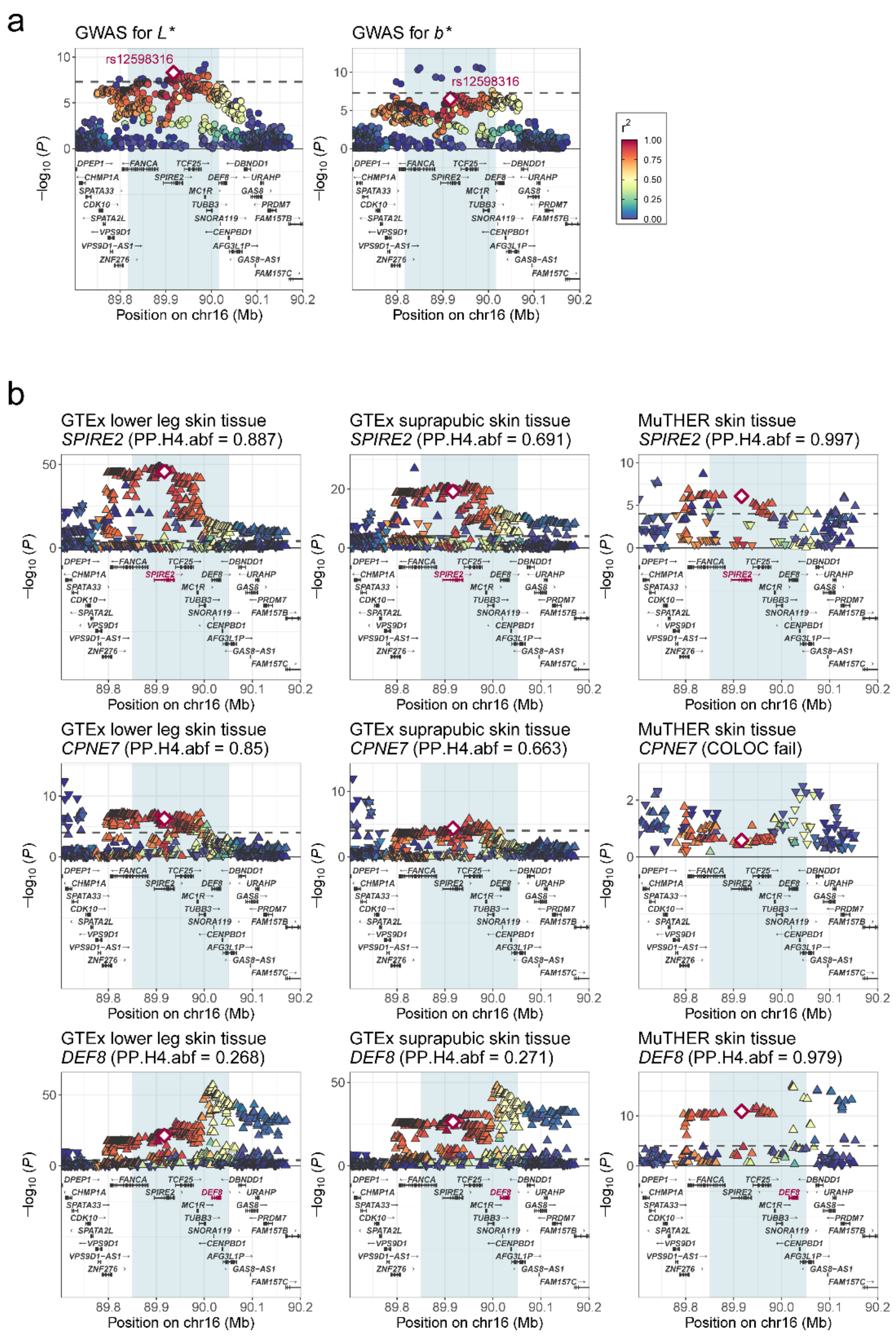

Supplementary Fig. S17. Regional plots of (**a**) GWAS results near *SPIRE2* and *MC1R* and (**b**) eQTL results of colocalized (PP.H4.abf > 0.7) genes near the corresponding loci, which are colored based on the lead variant on *SPIRE2*; ±250 kb from lead variants in each locus. Each dot represents a variant plotted as -$\log_{10}(P)$ on the *y*-axis against the corresponding variant position (Mb) on the *x*-axis and is colored according to linkage disequilibrium with the lead variant (rhombus). The blue-shaded region was used for colocalization analysis (±100 kb).

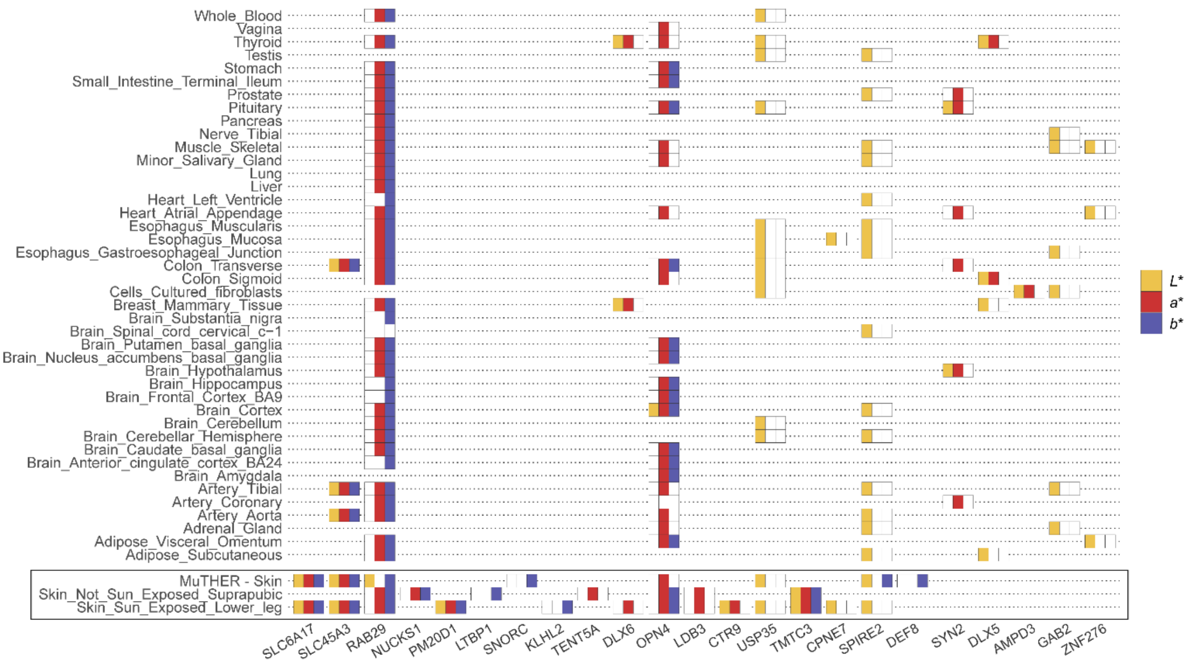

Supplementary Fig. S18. Colocalization results. Solid boxes indicate that eQTLs of gene (*x*-axis) expression in the tissue (*y*-axis) were colocalized (PP.H4 > 0.8) with GWAS for the color-corresponding phenotypes; yellow, red, and blue represent L^*, a^*, and b^*, respectively.

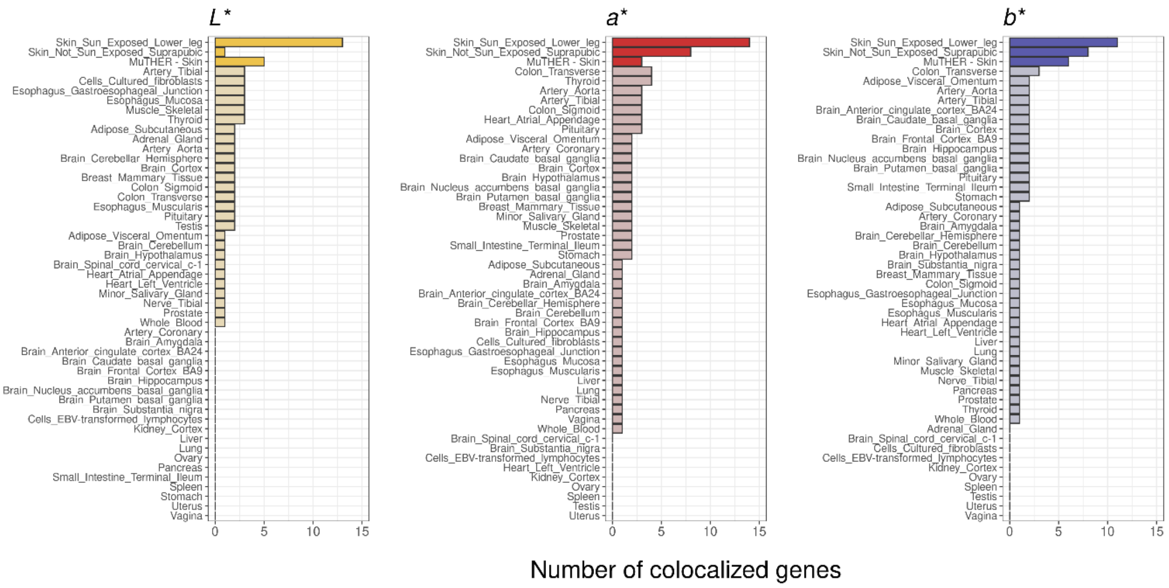

Supplementary Fig. S19. Number of colocalized genes (PP.H4 > 0.8) in each tissue. Each bar represents the number of colocalized genes (*x*-axis) in each tissue (*y*-axis). The skin tissues are highlighted in a darker color.

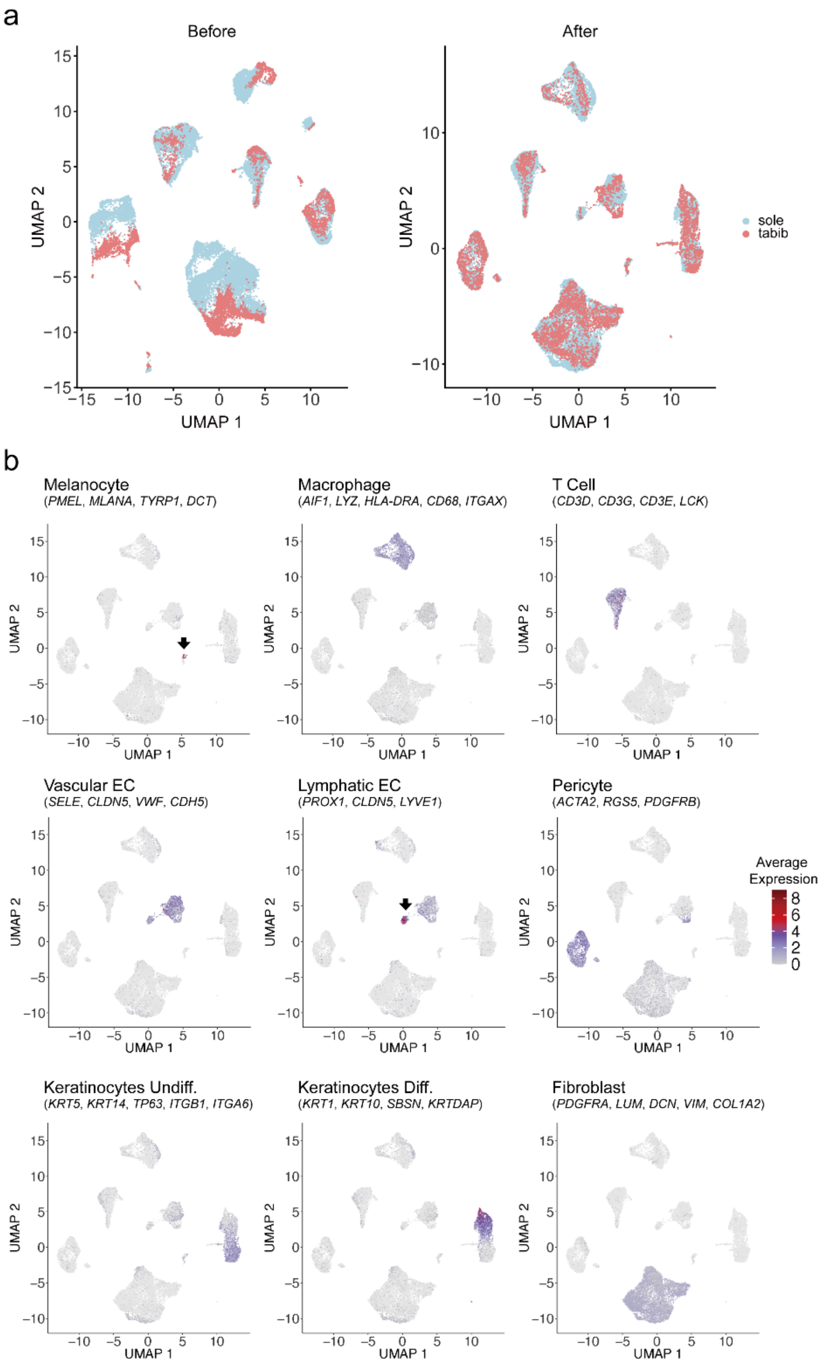

Supplementary Fig. S20. Batch effect correction for Harmony and gene expression patterns of skin cell-type markers. **a**, UMAP plots are presented for comparison before and after batch effect correction using Harmony. Red and blue points represent Sole et al. (2020), and Tabib et al. (2018) data, respectively. **b**, UMAP plots showing the gene expression patterns of well-known cell type markers with gene names in parentheses. Colors represent the average expression level of each cell type marker. Arrows indicate clusters with a small number of cells (melanocytes and lymphatic ECs). Abbreviations: Keratinocytes Diff., differentiated keratinocytes; Keratinocytes Undiff., undifferentiated keratinocytes; EC, endothelial cells.

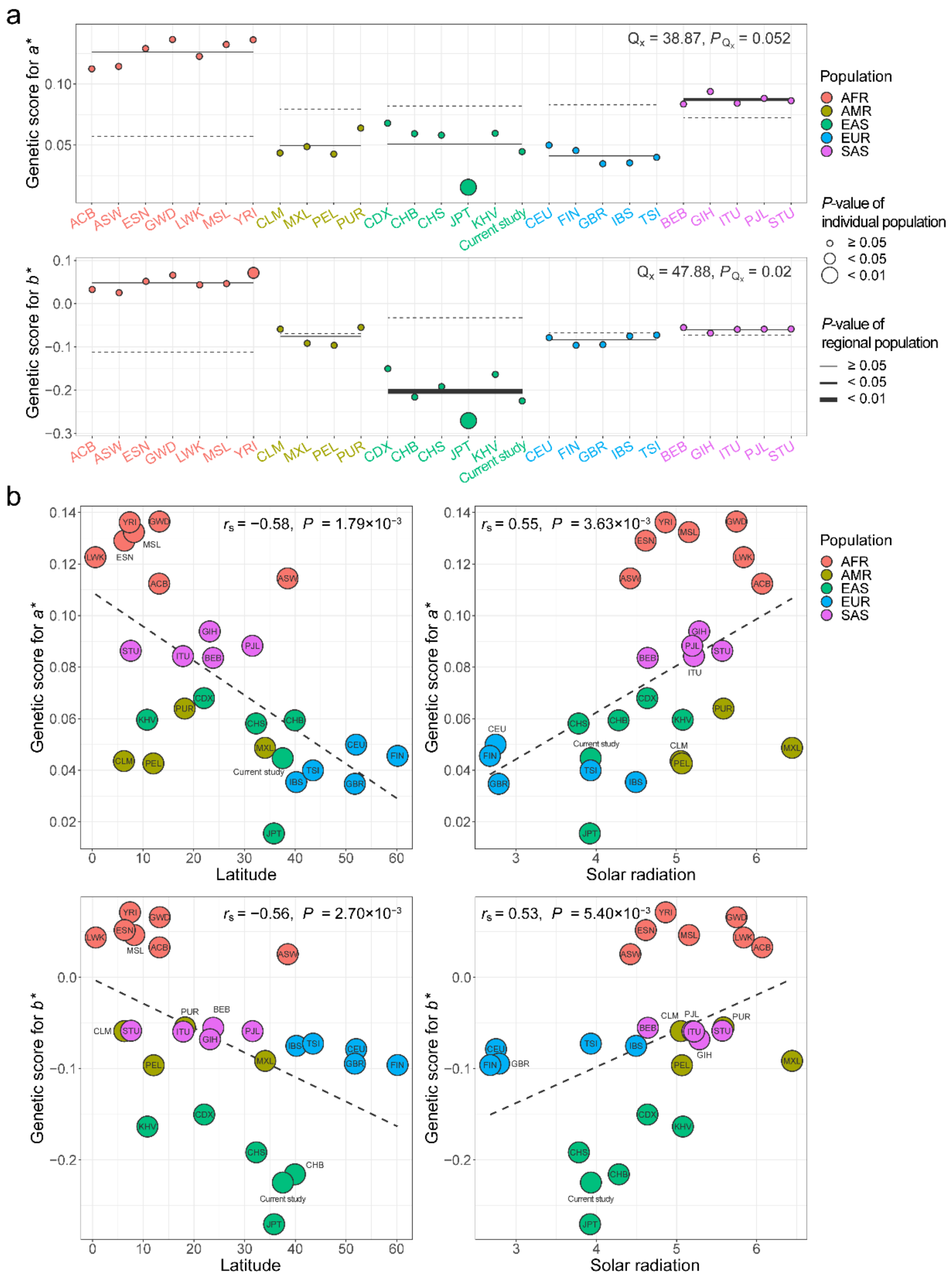

Supplementary Fig. S21. Signals of polygenic adaptation for b^* and a^* across the 1000 Genomes Project populations. **a**, Distribution of estimated genetic scores for b^* (top) and a^* (bottom) across the 1000 Genomes Project populations. Test statistics for the overdispersion of genetic values (Q_x) and *P*-values are presented at the top of each plot. **b**, Estimated genetic scores for a^* and b^* plotted against environmental factors: absolute latitude of each population (left) and annual solar radiation (right). The regression lines (dashed lines) show the linearity between the genetic score (*y*-axis) and environmental factors (*x*-axis). Spearman's correlation (r_s) and *P*-values are presented at the top of each plot. The *P*-value of Spearman's correlation coefficient was estimated under the null distribution of all possible permutations.

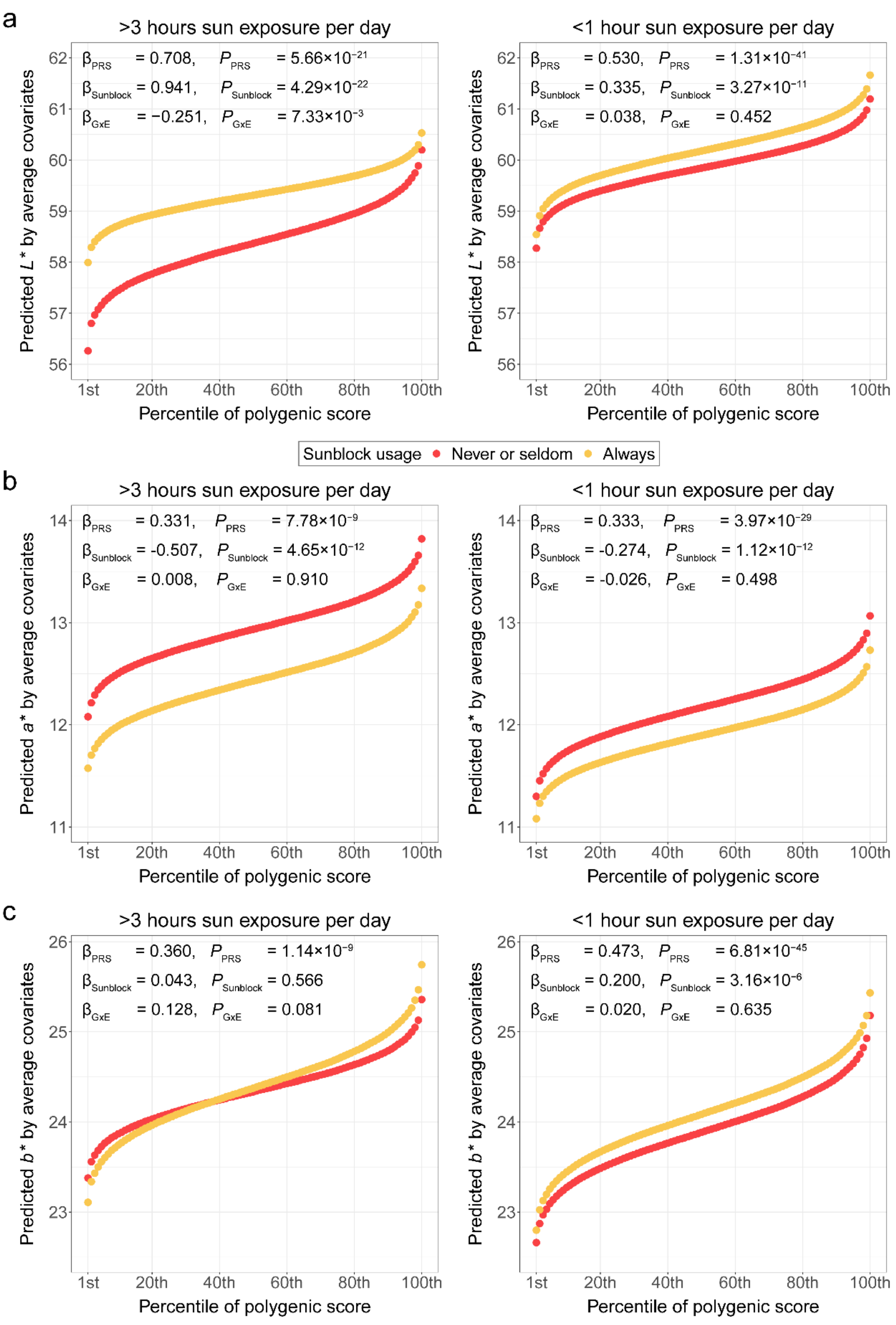

Supplementary Fig. S22. Interplay between polygenic score and sun exposure for (**a**) *L**, (**b**) *a**, and (**c**) *b**. Predicted value by average covariates in each percentile of polygenic score distribution for participants with never or seldom sunblock usage (red) and always sunblock usage (yellow) within each group divided by sun exposure per day: more than 3 hour (left) and less than 1 hour (right). The effect size ($\beta_{G\times E}$) and *P*-values ($P_{G\times E}$) of the interaction between the polygenic score and sunblock usage within each sun exposure group are presented at the top of each plot.

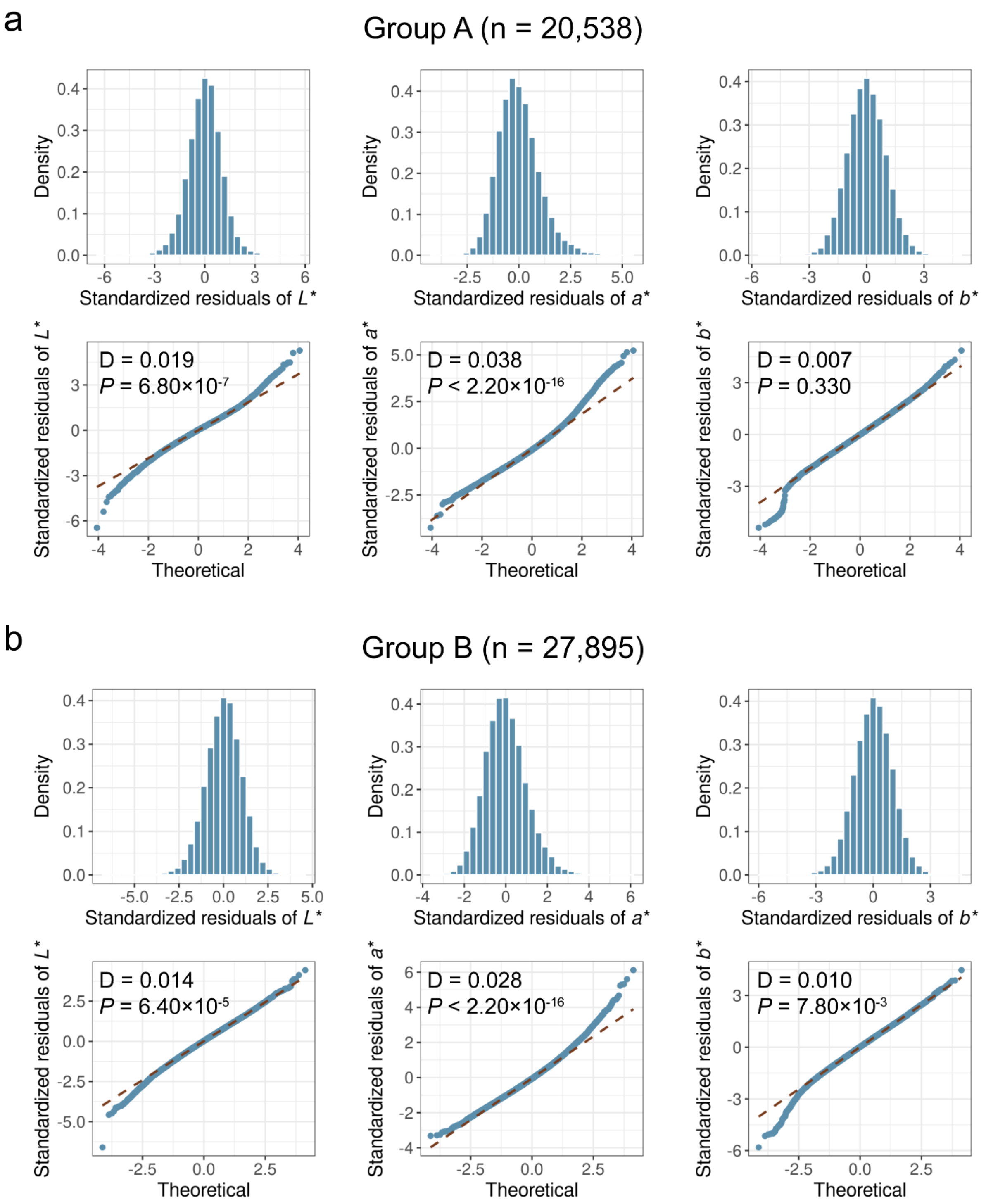

Supplementary Fig. S23. Normality of residuals for each skin color trait in a null model (a linear model with only covariates) assessed in each group (**a**) A and (**b**) B. Age, sex, sun-exposure variables, measurement month, genotyping batches, and the first 10 PCs of genetic ancestry were adjusted for the null model. Distribution of residuals (top) and quantile-quantile plots (bottom) were described with test statistic D and *P*-value of Kolmogorov-Smirnov test.

Software URLs utilized in this study

BOLT-LMM v.2.3.4, https://alkesgroup.broadinstitute.org/BOLT-LMM/BOLT-LMM_manual.html;

KING v.2.1, https://www.kingrelatedness.com;

SAIGE v.0.35.8, https://github.com/weizhouUMICH/SAIGE;

METAL (released on 2011-03-25), https://genome.sph.umich.edu/wiki/METAL;

PLINK v.1.90, https://www.cog-genomics.org/plink;

Eagle v.2.4.1, https://alkesgroup.broadinstitute.org/Eagle;

Minimac v.4, https://github.com/statgen/Minimac4;

GCTA v.1.91.2, https://yanglab.westlake.edu.cn/software/gcta/#Overview;

VEP v.98, https://asia.ensembl.org/info/docs/tools/vep/index.html;

POLMM (released on 2022-08-26), https://wenjianbi.github.io/grab.github.io;

DEPICT v.1.1, https://github.com/perslab/depict;

GARFIELD v.2, https://annahutch.github.io/PhD/garfield.html;

Polygenic adaptation (released on 2014-12-21), https://github.com/jjberg2/PolygenicAdaptationCode;

PRS-CS (released on 2021-06-04), https://github.com/getian107/PRScs;

coloc v5.1.1, https://chrlswallace.github.io/coloc/articles/a01_intro.html;

Seurat v.3.2.3, https://satijalab.org/seurat.
