# Supplementary material for: Mapping and annotating genomic loci to prioritize genes and implicate distinct polygenic adaptations for skin color
Source: Nat Commun. 2024 Jun 7;15:4874. doi: 10.1038/s41467-024-49031-4 (PMC11161515; doi:10.1038/s41467-024-49031-4)
Supplement: Supplementary file 3 — Description of Additional Supplementary Files [file 41467_2024_49031_MOESM3_ESM.pdf]

Description of Additional Supplementary Files

File Name: Supplementary Data 1
Description: Summary of previously reported genetic studies on skin color.

File Name: Supplementary Data 2
Description: Characteristics of the study participants in discovery set.

File Name: Supplementary Data 3
Description: The interplay between sun exposure variable and age group on skin color.

File Name: Supplementary Data 4
Description: Detailed summary statistics for the 26 lead variants in the discovery and replication GWAS.

File Name: Supplementary Data 5
Description: Independent nonsynonymous variants associated with skin color were identified from a meta-analysis of GWAS ($P < 5.44e-06$).

File Name: Supplementary Data 6
Description: Characteristics of the study participants in the replication set.

File Name: Supplementary Data 7
Description: Characteristics of the study participants in the cross-validation group.

File Name: Supplementary Data 8
Description: The power-adjusted transferability (PAT) ratio of the lead variants in the replication cohort and 10-fold cross-validation of GWAS.

File Name: Supplementary Data 9
Description: SNP-based heritability estimates of female subgroups according to age and partitioned heritability by LD quartiles and MAF quintiles.

File Name: Supplementary Data 10
Description: Functional enrichment analysis using DEPICT at skin color trait-associated loci.

File Name: Supplementary Data 11
Description: Colocalization between GWAS results and eQTL in multiple tissues ($PP.H4 > 0.8$ were considered as colocalized).

File Name: Supplementary Data 12
Description: Single-cell level gene expression of CIE LAB value-associated genes.

File Name: Supplementary Data 13
Description: Worldwide allele frequency of GWAS lead variants, absolute latitude, and mean annual solar radiation.

File Name: Supplementary Data 14
Description: Characteristics of the unrelated study participants by polygenic score group.
