# Supplementary material for: Mapping and annotating genomic loci to prioritize genes and implicate distinct polygenic adaptations for skin color
Source: Nat Commun. 2024 Jun 7;15:4874. doi: 10.1038/s41467-024-49031-4 (PMC11161515; doi:10.1038/s41467-024-49031-4)
Supplement: Supplementary file 5 — Reporting Summary [file 41467_2024_49031_MOESM5_ESM.pdf]

Corresponding author(s): Nae Gyu Kang
Hong-Hee Won

Last updated by author(s): Apr 26, 2024

Reporting Summary

Nature Portfolio wishes to improve the reproducibility of the work that we publish. This form provides structure for consistency and transparency in reporting. For further information on Nature Portfolio policies, see our Editorial Policies and the Editorial Policy Checklist.

Statistics

For all statistical analyses, confirm that the following items are present in the figure legend, table legend, main text, or Methods section.

n/a	Confirmed	
☐	☒	The exact sample size (*n*) for each experimental group/condition, given as a discrete number and unit of measurement
☐	☒	A statement on whether measurements were taken from distinct samples or whether the same sample was measured repeatedly
☐	☒	The statistical test(s) used AND whether they are one- or two-sided<br>*Only common tests should be described solely by name; describe more complex techniques in the Methods section.*
☐	☒	A description of all covariates tested
☐	☒	A description of any assumptions or corrections, such as tests of normality and adjustment for multiple comparisons
☐	☒	A full description of the statistical parameters including central tendency (e.g. means) or other basic estimates (e.g. regression coefficient) AND variation (e.g. standard deviation) or associated estimates of uncertainty (e.g. confidence intervals)
☐	☒	For null hypothesis testing, the test statistic (e.g. *F*, *t*, *r*) with confidence intervals, effect sizes, degrees of freedom and *P* value noted<br>*Give P values as exact values whenever suitable.*
☒	☐	For Bayesian analysis, information on the choice of priors and Markov chain Monte Carlo settings
☒	☐	For hierarchical and complex designs, identification of the appropriate level for tests and full reporting of outcomes
☐	☒	Estimates of effect sizes (e.g. Cohen's *d*, Pearson's *r*), indicating how they were calculated

Our web collection on statistics for biologists contains articles on many of the points above.

Software and code

Policy information about availability of computer code

Data collection	In this study, 52,712 participants of East Asian ancestry with no severe medical conditions at the time of recruitment were recruited through offline cosmetics shops in 2018. The Institutional Review Board (IRB) of the LG Household & Healthcare Research Center approved this study (IRB Nos. 2017-PB-0001 and 2018-PB-001). To measure skin color, facial images were obtained using a Janus III system under normal light conditions (PIE Inc., Suwon, Korea). Skin color image analysis was performed using an internal algorithm of the measuring instrument, which converted the images into numerical values (CIE LAB values: L*, a*, and b*).
Data analysis	BOLT-LMM v.2.3.4, https://alkesgroup.broadinstitute.org/BOLT-LMM/BOLT-LMM_manual.html;<br>KING v.2.1, https://www.kingrelatedness.com;<br>SAIGE v.0.35.8, https://github.com/weizhouUMICH/SAIGE;<br>METAL (released on 2011-03-25), https://genome.sph.umich.edu/wiki/METAL;<br>PLINK v.1.90, https://www.cog-genomics.org/plink;<br>Eagle v.2.4.1, https://alkesgroup.broadinstitute.org/Eagle;<br>Minimac v.4, https://github.com/statgen/Minimac4;<br>GCTA v.1.91.2, https://yanglab.westlake.edu.cn/software/gcta/#Overview;<br>VEP v.98, https://asia.ensembl.org/info/docs/tools/vep/index.html;<br>POLMM (released on 2022-08-26), https://wenjianbi.github.io/grab.github.io;<br>DEPICT v.1.1, https://github.com/perslab/depict;<br>GARFIELD v.2, https://annahutch.github.io/PhD/garfield.html;<br>Polygenic adaptation (released on 2014-12-21), https://github.com/jjberg2/PolygenicAdaptationCode;<br>PRS-CS (released on 2021-06-04), https://github.com/getian107/PRScs;

coloc v5.1.1, https://chrlswallace.github.io/coloc/articles/a01_intro.html;
Seurat v.3.2.3, https://satijalab.org/seurat.

For manuscripts utilizing custom algorithms or software that are central to the research but not yet described in published literature, software must be made available to editors and reviewers. We strongly encourage code deposition in a community repository (e.g. GitHub). See the Nature Portfolio guidelines for submitting code & software for further information.

Data

Policy information about availability of data

All manuscripts must include a data availability statement. This statement should provide the following information, where applicable:

- Accession codes, unique identifiers, or web links for publicly available datasets
- A description of any restrictions on data availability
- For clinical datasets or third party data, please ensure that the statement adheres to our policy

The genotype and phenotype data of East Asian participants in the analysis were collected by Migenstory, a subsidiary of LG Household & Healthcare. This individual-level genotype and phenotype data are protected and are not available due to data privacy laws. The full summary statistics of GWAS for L* (luminance), a* (red/green component), and b* (yellow/blue component) in 48,433 East Asians are publicly available at the NHGRI-EBI GWAS Catalog (https://www.ebi.ac.uk/gwas/downloads) with accession numbers GCST90320257, GCST90320258, and GCST90320259, respectively. The summary statistics of associations of variants in chromosome X with L* (luminance), a* (red/green component), and b* (yellow/blue component) in 42,770 East Asian females are publicly available at the NHGRI-EBI GWAS Catalog with accession numbers GCST90320260, GCST90320261, and GCST90320262, respectively. The accession numbers of GWAS Catalog will be available before publication. The UKBB genotype and epidemiologic data are available by requesting access on the UKBB homepage (https://www.ukbiobank.ac.uk/). The GTEx data are publicly available upon reasonable application (http://www.gtexportal.org/home/datasets). The MuTHER data are publicly available upon reasonable application (http://www.muther.ac.uk/Data.html). The scRNA-seq data were collected from the database in Gene Expression Omnibus (https://www.ncbi.nlm.nih.gov/geo/query/acc.cgi?acc=GSE130973) and University of Pittsburgh (https://dom.pitt.edu/wp-content/uploads/2018/10/Skin_6Control_rawUMI.zip). The surface solar radiation data from January 1984 to December 2022 were collected from the NASA POWER project (https://power.larc.nasa.gov/data-access-viewer). The 1000 Genomes Project phase 3 data are publicly available (https://www.internationalgenome.org/data).

Research involving human participants, their data, or biological material

Policy information about studies with human participants or human data. See also policy information about sex, gender (identity/presentation), and sexual orientation and race, ethnicity and racism.

Reporting on sex and gender	We used sex as a covariate to adjust our analyses. In total, 5,663 males (11.7%) and 42,770 (88.3%) females were included in the East Asian GWAS. There is no mention of gender in this study. The interplay between polygenic scores and environmental variables and associations on the X chromosome were analyzed only in female samples to minimize confounding effects.
Reporting on race, ethnicity, or other socially relevant groupings	A GWAS was conducted for a total of 48,433 individuals of East Asian ancestry. Polygenic score was assessed for the GWAS samples and 2,332 individuals of East Asian ancestry from the UK Biobank. Ancestral background of study participants were investigated using questionnaire. Confounding variables, including the ancestral background, were adjusted in all of the association models in this study.
Population characteristics	Analyses used age, sex, sun-exposure variables, measurement month, genotyping batch, and principal components from the genetic data as covariates to control for the population structure. More characteristics of study samples were described in Supplementary Tables S2, S6, S7, and S14.
Recruitment	In the discovery study, 52,712 participants of East Asian ancestry with no severe medical conditions at the time of recruitment were recruited through offline cosmetics shops in 2018. Participants were divided into two groups based on camera resolution: Group A, in which the skin color of 23,454 participants was measured using 18-megapixel images, and Group B, in which the skin color of 29,258 participants was measured using 24.2-megapixel images. There was no significant difference between the two groups, and each group was meta-analyzed after GWAS analysis.
Ethics oversight	This study has been approved by LG Household & Healthcare (IRB No. 2017-PB-0001 and 2018-PB-001).

Note that full information on the approval of the study protocol must also be provided in the manuscript.

Field-specific reporting

Please select the one below that is the best fit for your research. If you are not sure, read the appropriate sections before making your selection.

☒ Life sciences ☐ Behavioural & social sciences ☐ Ecological, evolutionary & environmental sciences

For a reference copy of the document with all sections, see nature.com/documents/nr-reporting-summary-flat.pdf

Life sciences study design

All studies must disclose on these points even when the disclosure is negative.

Sample size	The total sample size was 48,433 East Asians, consisting of 5,663 males (11.7%) and 42,770 (88.3%) females. We tried to analyze as many samples as possible rather than statistical sample size calculation. The total sample size of the current study is the largest sample size to date among the East Asian studies, and give sufficient statistics for the effect sizes of associations.

Data exclusions	We excluded both unqualified samples and variants based on elaborate quality control procedures. Detailed information was described in the method section and Supplementary Notes.
Replication	The association between the lead variants and skin color traits was examined in 4,992 individuals (10.3% of the discovery cohort) who were externally independent of the discovery cohort. In the replication GWAS, the effect sizes of the lead variants were highly consistent with those in the discovery GWAS, although the significance level of the associations was limited. To assess the replicability under comparable sample sizes, we conducted 10-fold cross-validation in the discovery set (4,843–4,846 individuals in a validation set). The power-adjusted transferability (PAT) ratio, determined by dividing the observed number by the expected number of nominally significant ($P < 0.05$) loci (Nature Communications, 2022), of cross-validation was similar to that in the replication analysis. The association of the lead variants derived from the GWAS was partially replicated in 10% of individuals of the discovery cohort, although the effect sizes of the discovery result were consistent with the replication result. The identification of previously unreported loci associated with skin color in the current study might be attributable to the larger sample size than previous research in non-European populations.
Randomization	Because this study is an observational GWAS and not a clinical experimental study, randomization was not required during the discovery phase. We identified genetic variants associated with skin color by adjusting for covariates in the model instead of randomization.
Blinding	Blinding to group allocation was not relevant to this study. Study participants were recruited through offline cosmetics shops and skin color was objectively measured as 'quantitative trait'.

Reporting for specific materials, systems and methods

We require information from authors about some types of materials, experimental systems and methods used in many studies. Here, indicate whether each material, system or method listed is relevant to your study. If you are not sure if a list item applies to your research, read the appropriate section before selecting a response.

Materials & experimental systems

n/a	Involved in the study
☒	☐ Antibodies
☒	☐ Eukaryotic cell lines
☒	☐ Palaeontology and archaeology
☒	☐ Animals and other organisms
☒	☐ Clinical data
☒	☐ Dual use research of concern
☒	☐ Plants

Methods

n/a	Involved in the study
☒	☐ ChIP-seq
☒	☐ Flow cytometry
☒	☐ MRI-based neuroimaging

Plants

Seed stocks	*Report on the source of all seed stocks or other plant material used. If applicable, state the seed stock centre and catalogue number. If plant specimens were collected from the field, describe the collection location, date and sampling procedures.*
Novel plant genotypes	*Describe the methods by which all novel plant genotypes were produced. This includes those generated by transgenic approaches, gene editing, chemical/radiation-based mutagenesis and hybridization. For transgenic lines, describe the transformation method, the number of independent lines analyzed and the generation upon which experiments were performed. For gene-edited lines, describe the editor used, the endogenous sequence targeted for editing, the targeting guide RNA sequence (if applicable) and how the editor was applied.*
Authentication	*Describe any authentication procedures for each seed stock used or novel genotype generated. Describe any experiments used to assess the effect of a mutation and, where applicable, how potential secondary effects (e.g. second site T-DNA insertions, mosiacism, off-target gene editing) were examined.*
